# Attitudes towards bystander cardiopulmonary resuscitation: Results from a cross-sectional general population survey

**DOI:** 10.1371/journal.pone.0193391

**Published:** 2018-03-07

**Authors:** Fiona Dobbie, Anne Marie MacKintosh, Gareth Clegg, Rebecca Stirzaker, Linda Bauld

**Affiliations:** 1 Institute for Social Marketing, Faculty of Health Sciences and Sport, University of Stirling, Stirling, United Kingdom; 2 The University of Edinburgh, Resuscitation Research Group, Edinburgh, United Kingdom; 3 Heriot Watt University, School of Social Sciences, Edinburgh, United Kingdom; Azienda Ospedaliero Universitaria Careggi, ITALY

## Abstract

Survival from out-of-hospital cardiac arrest (OHCA) varies across the developed world. Although not all OHCA are recoverable, the survival rate in Scotland is lower than in comparable countries, with higher average survival rates of 7.9% in England and 9% across Europe. The purpose of this paper is to explore the barriers, facilitators and public attitudes to administering bystander cardiopulmonary resuscitation (CPR) which could inform future policy and initiatives to improve the rate of bystander CPR. Data was collected via a cross-sectional general population survey of 1027 adults in Scotland. 52% of respondents had been trained in CPR. Of those who were not trained, two fifths (42%) expressed a willingness to receive CPR training. Fewer than half (49%) felt confident administering CPR, rising to 82% if they were talked through it by a call handler. Multivariate analyses identified that people in social grade C2DE were less likely than those in social grade ABC1 to be CPR trained and less confident to administer CPR if talked through by a call handler. The older a person was, the less likely they were to be CPR trained, show willingness to be CPR trained or be confident to administer bystander CPR with or without instruction from an emergency call handler. These findings are particularly relevant considering that most OHCA happen in the homes of older people. In a developed country such as Scotland with widely available CPR training, only half of the adult population reported feeling confident about administering bystander CPR. Further efforts tailored specifically for people who are older, unemployed and have a lower social grade are required to increase knowledge, confidence and uptake of training in bystander CPR.

## Introduction

Survival from out-of-hospital cardiac arrest (OHCA) varies across the developed world. In 2013 Seattle, USA, had one of the best OHCA survival to discharge from hospital rates at 22% [1]. In contrast resuscitation is attempted for approximately 3,000 adults who experience out-of-hospital cardiac arrest (OHCA) each year in Scotland with only 6% surviving to hospital discharge [1]. Although not all OHCA are recoverable, the survival rate in Scotland is lower than in comparable countries, with higher average survival rates of 7.9% in England [2] and 9% across Europe [3]. There is also considerable regional variability in survival outcomes with cities like Stavanger in Norway reporting survival rates as high as 25% [3,4]. While these figures should be interpreted with caution (due to variation in the way data is presented) there is a clear public health policy agenda to improve survival after OHCA in Scotland.

The experience of other national OHCA survival programmes has shown that increasing bystander CPR improves overall survival [5]. In Sweden a CPR training strategy resulted in three million people being trained in CPR (population 9.5 million) during the last three decades [6]. By 2011 the CPR rate had risen from 31% to over 70%—amongst the highest in the world—with a parallel increase in OHCA survival to one month from 5% in 1992 to 11% in 2011 [7].

In 2015 Scotland launched a national strategy for out-of-hospital cardiac arrest with the ambition that by 2020 Scotland becomes an international leader in OHCA outcomes [1]. The overall aim is to save an additional 1,000 lives by 2020. Crucial to achieving this is to increase rates of bystander CPR—currently estimated to be around 50% [8].

To help meet this aim a cross-sectional survey of the Scottish population was conducted in 2015 to explore public attitudes, awareness and perception of bystander CPR. This paper presents survey findings focusing on the current prevalence of CPR training and modifiable factors (such as barriers to administering bystander CPR) which could inform future policy and initiatives to improve the rate of bystander CPR.

## Methods

Data collection took place between 5^th^-18^th^ August 2015 via the Scottish Opinion Survey (SOS). The SOS is an omnibus survey administered by social research agency, KANTAR Public UK (formerly TNS-BMRB). Survey questions and pre-coded answer options were developed by the research team after reviewing the literature. These were then reviewed and discussed with the project steering group and further refinements were made to reach the final set of questions.

Random household location quota sampling was used to generate a sample of 1027 adults aged 16 and over across Scotland, selected by age, gender, working status and number of children in the household. Weights were applied to the sample to be representative of the adult population. Interviews were conducted face-to-face, in the home (one interview per household), by trained market research interviewers via a computer programme called CAPI (Computer Assisted Personal Interviewing). Verbal consent was obtained before data collection took place. Ethical approval was obtained from the University of Stirling’s School of Health Sciences committee prior to data collection.

Demographic information included age, gender, working status and social grade. Social grade was determined using the National Readership Survey (NRS) based on the occupation of the chief income earner in the household. Social grade ‘ABC1’ includes professional, managerial and non-manual occupations, while ‘C2DE’ includes manual and unskilled occupations and the long-term unemployed.

### Analysis

Data were analysed using SPSS version 21. Descriptive data were weighted to match the adult population in terms of age, sex and social grade. Weighting adjusts the results so that groups that are under-represented in the sample are given a weight greater than 1 while those who are over-represented in the sample are assigned weights lower than 1. Percentages presented in the results section are based on the weighted sample. Chi-square tests were run to examine responses by gender, age, social grade and whether or not participants were trained in CPR. Logistic regression was used to enable assessment of the association between multiple demographic variables and likelihood of administering CPR in a hypothetical situation. Control variables were entered using the enter method, a forced entry approach which means variables are put into and remain in the model regardless of whether or not they have a significant association with the outcome variable. Age was entered as a categorical variable, with ‘35–44’ taken as the reference category. When examining confidence in, and likelihood of administering, CPR the analyses also controlled for previous training in CPR.

## Results

### CPR training

Just over half (52%, n = 536) of respondents had been trained in CPR (Table 1), but two fifths (44%) had received this training over five years ago. The likelihood of having been trained in CPR differed by age (p<0.001) and social grade (p<0.01) (Table 1). For example, while 60% of 35–44 year olds had been trained, only 35% of those aged 65+ had been trained in CPR. Respondents with professional, managerial and non-manual occupations (ABC1) were more likely to have been trained than those in manual, unskilled occupations and the long-term unemployed (C2DE) (57% ABC1 v 48% C2DE, p<0.01). Likelihood of being trained in CPR did not differ by gender.

**Table 1:** Training in CPR and confidence in administering CPR: by gender, age, social grade and trained status.

Base: *All respondents (1027)*	Total	Gender		Age							Social Grade		Trained Status	
		Male	Female	16–17	18–24	25–34	35–44	45–54	55–64	65+	ABC1	C2DE	Trained	Not trained
*N*	*1027*	*493*	*534*	*26*	*122*	*161*	*167*	*184*	*156*	*211*	*512*	*515*	*536*	*488*
	%	%	%	%	%	%	%	%	%	%	%	%	%	%
Trained in CPR	52	53	52	46	55	59	60	56	54	35***	57	48**	100	0
Would like to be trained in CPR	42	39	45	57	52	57	58	45	37	23***	46	39	N/A	42
Feel confident about administering CPR	49	52	46	44	47	50	55	59	47	36***	48	49	72	23***
Feel confident about administering CPR if talked through by a call handler	82	82	81	85	81	84	86	89	83	70***	86	78**	93	70***

*** p<0.001;

** p<0.01;

* p<0.05

### Attitudes towards receiving CPR training

Of those who were not CPR trained, 42% (n = 208) said they would like to receive training (Table 1). Once again age was an important factor with older respondents less willing to be trained in CPR (p<0.001). For example, 58% of 35–44 year olds said they would like to be trained in CPR, which compares with just 37% of 55–64 year olds and 23% of those aged 65+.

### Confidence to administer CPR

Confidence to administer CPR differed by age (p<0.001) and training status (p<0.001). For example, while 55% of 35–44 year olds indicated they would be confident administering CPR, only 36% of those aged 65+ indicated they would feel confident (Table 1). The majority (72%) of those who had been trained said they would be confident while 23% of those who had not been trained said they would be confident administering it.

### Confidence to administer CPR with call handler instruction

The majority of respondents, 82% (n = 839) reported that they would feel confident administering CPR if an emergency call handler talked them through it (Table 1). Confidence differed by age (p<0.001). For example, while 86% of 35–44 year olds and 89% of 45–54 year olds said they would be confident, only 70% of those aged 65+ said they would be confident. Respondents in social grade ABC1 were more likely to feel confident than those in social grade C2DE (86% ABC1 v 78% C2DE, p<0.01). The vast majority of those who had been trained said they would be confident compared with 70% among those not trained (p<0.001).

### Likelihood of administering CPR

When presented with the following hypothetical scenario—*I’d like you to imagine that you are walking down the street and you see an average person collapse*. *They are unconscious*, *not breathing and have no pulse*. *If you were the only person there*, *how likely or unlikely is it that you would give this person CPR*?—the majority (72%) (n = 742) of respondents were either extremely or somewhat likely to administer CPR. Influencing factors were (Table 2): being trained in CPR, with respondents who were trained in CPR were more than four times as likely as untrained respondents to administer CPR (AOR 4.745, 95% CI 3.470 to 6.490, p<0.001); gender, with men were more likely to administer CPR (AOR 1.396, 95% CI 1.041 to 1.873, p<0.05) and; age. The youngest respondents (16 to 17 year olds) were less likely than 35 to 44 year olds to administer CPR (AOR 0.342, 95% CI 0.122 to 0.960, p<0.05). Similarly, the oldest age group (aged 65+) were less likely than 35 to 44 year olds to consider it likely that they would give CPR (AOR 0.459, 95% CI 0.267 to 0.787, p<0.01). There was no difference by social grade, or working status.

**Table 2:** Likelihood of administering CPR.

	N	Adj OR*	95% CI Lower	95% CI Upper	P
**Trained in CPR**					
Not trained	519	ref			
Trained	506	4.76	3.48	6.50	<0.001
**Age**					
35–44	144	ref			0.002
16–17	21	0.33	0.12	0.94	0.037
18–24	93	0.83	0.42	1.61	0.576
25–34	136	0.75	0.41	1.37	0.356
45–54	177	0.91	0.51	1.61	0.747
55–64	133	0.56	0.31	1.01	0.053
65+	321	0.44	0.27	0.72	0.001
**Gender**					
Female	550	ref			
Male	475	1.40	1.05	1.88	0.023
**Social Grade**					
C2DE	548	ref			
ABC1	477	1.07	0.80	1.44	0.640

Dependent Variable: Whether would be likely to give someone CPR, 1 = (Would be likely (719), 0 = Not (306). Test of model coefficients: χ^2^ = 154.359 df = 9, p<0.001. Nagelkerke R^2^ = 0.198. Hosmer & Lemeshow χ^2^ = 4.489, df = 8, p = 0.810.

* adjusted for all other variables in the model

Adjusted OR, adjusted odds ratio; ref, reference category; 95% CI, 95% confidence interval

### Perceived barriers to administering CPR

When respondents were asked to select from a list of pre-specified (Table 3) reasons which may explain why they would not administer CPR the most common reasons were fear about causing injury/making things worse (22%) (n = 222), visible signs of vomit/blood (19%) (n = 197), lack of skills (19%) (n = 192), lack of confidence (15%) (n = 150) or concern that the person might be a drug user (16%) (n = 162). One third (33%) (n = 343) said none of the reasons were applicable to them.

**Table 3:** Reasons CPR would not be attempted (n = 1027).

			Trained		Not trained		P
	N	%	N	%	N	%	
Fear I may catch a disease	100	10	52	10	48	10	0.942
Person looks dirty	55	5	27	5	28	6	0.62
Visible sign of vomit/blood	197	19	124	23	72	15	0.001
Smell of alcohol	105	10	62	12	44	9	0.181
Person is a drug user	162	16	47	18	65	13	0.038
Do not have the skills to give CPR	192	19	22	4	17	35	0.001
Do not have the confidence to give CPR	150	15	37	7	112	23	0.001
Fear of being sued	85	8	49	9	36	7	0.311
Fear about causing injury / making things worse	222	22	93	17	129	26	0.001
I do not want to give mouth to mouth resuscitation	67	7	34	6	33	7	0.787
I do not know the person	22	2	9	2	13	3	0.287
Not sure if they need CPR	147	14	50	9	95	20	0.001
None	343	33	222	41	120	25	0.001

Amongst respondents who were not CPR trained the most common reason was not having the skills to give CPR (35%) (n = 17) which compares with just 4% (n = 22) of respondents who were trained. Visible signs of vomit/blood were cited as the main reason for not administering CPR amongst those who were CPR trained (23%) (n = 124), which compares with (15%) (n = 72) of respondents who were not trained.

## Discussion

The purpose of this cross-sectional survey was twofold. First, baseline data on the number of people trained in CPR has been collected to help monitor progress of the OHCA strategy in Scotland. Second, in order to inform future policy and initiatives to improve the rate of bystander CPR, the barriers and facilitators to administering bystander CPR were explored.

Despite over half (52%) of respondents being trained in CPR, two fifths (44%) had received this training over five years ago. Among those who were not trained, 42% expressed a willingness to be trained in CPR. One fifth of the whole sample (21%) would not know if CPR was required and a further 50% would not feel confident administering CPR. These findings suggest that if Scotland is to become an international leader in OHCA outcomes more needs to be done to improve levels of confidence to administer bystander CPR if required, especially among older age groups and people with lower social grade.

However, respondents did show a willingness to administer bystander CPR if they were the only person present (72% of respondents said they would be extremely or somewhat likely to do so). This is surprising given the low level of confidence, but may be explained by results from other research. Johnston et al (2003) found that CPR by an individual was more likely where they thought no one else was present and less likely where they were not alone. This could be due to the *Bystander Effect*: the diffusion of responsibility when there are many people present in an emergency situation [9].

Reasons for the lack of confidence to administer bystander CPR identified in our sample were congruent with existing evidence from a number of countries, which points to a range of individual and environmental factors [10–14]. Individual factors focus on the perceptions, knowledge and CPR experience of the person witnessing a cardiac arrest. One of the most common reasons for unwillingness to intervene in OHCA is fear of causing more harm [12,13]. This was the main barrier from our survey cited by 22% of respondents. Moreover, there is a perception that this fear will be exacerbated in an emergency situation through panic [10–14]. Several studies note that bystanders are more willing to perform CPR on children and young adults than on older people or socially excluded groups (i.e. intravenous drug users or homeless people) and can be put off by physical presentation; e.g. presence of vomit or dentures, for example [11, 15–17]. Our results are consistent with this literature with visible signs of vomit/blood, or a concern that the victim may be a drug user being some of the most common barriers. It is notable that when respondents were asked to identify reasons why they may not administer bystander CPR, 33% said that none of the pre specified reasons were relevant to them. This suggests that further qualitative research is required to enhance understanding of the potential barriers to administering bystander CPR.

Concerns about legal action (such as being sued) for attempting CPR did not feature strongly in our sample, with just 8% citing this as a barrier. This is consistent for countries without a strong personal litigation culture such as Scotland, but has been identified as a barrier in countries that do, such as the USA [14,18].

The more economically deprived an area is, the higher the rate of cardiac arrest—but survival rates are lower [19]. However studies have shown that, currently, bystander CPR intervention is higher in wealthier than deprived areas [19,20]. This suggests the need for optimal bystander CPR intervention in more deprived areas. In Scotland, a similar social gradient in OHCA and CPR response has been found. As levels of deprivation rise so do rates in OHCA, but bystander CPR interventions fall [1]. Our multivariate analyses identified that employment status and social grade (which influence levels of deprivation), along with age, were significant factors affecting bystander CPR. People in social grade C2DE were less likely than those in social grade ABC1 to be CPR trained and less confident to administer CPR if talked through by a call handler. Finally, the older a person was, the less likely they were to be CPR trained, show willingness to be CPR trained or be confident to administer bystander CPR. These findings are particularly relevant considering that most OHCA happen in the homes of older people [21], and they provide a clear indication of where future efforts need to be targeted if rates of bystander CPR are to be improved.

From a policy perspective there is a need for more tailored and targeted interventions to encourage CPR training which has been linked with improving confidence in CPR. As this increases, so does the likelihood of providing emergency aid in an OHCA [17,22]. Our findings suggest that priority groups are people who are not working, in a lower social grade and the elderly.

Our study has limitations. First, the pre-coded answer options for the survey may have influenced responses and there may have been factors influencing responses which we did not capture in our questions. Second, our results were limited to a cross-sectional survey from a random location quota sample which is a non-probability sampling approach. As a result, no detail is available on response rate and the results are not necessarily generalisable to the wider population. Finally, we were not able to add to the literature around optimal approaches to engage local communities to take part in CPR training and build confidence. Instead, this is a current focus for future work in a new study funded by the Chief Scientist’s Office of the Scottish Government.

## Conclusions

In a developed country such as Scotland with widely available CPR training, only half of the adult population reported feeling confident about administering bystander CPR. Further efforts tailored specifically for people who are older, unemployed and have a lower social grade are required to increase knowledge, confidence and uptake of training in bystander CPR in order to improve cardiac arrest survival.
